# Assessing Hotel Employee Knowledge on Risk Factors and Risk Management Procedures for Microbial Contamination of Hotel Water Distribution Systems

**DOI:** 10.3390/ijerph18073539

**Published:** 2021-03-29

**Authors:** Brandon Leftwich, Samuel T. Opoku, Jingjing Yin, Atin Adhikari

**Affiliations:** 1 Department of Health Policy and Community Health, Jiann-Ping Hsu College of Public Health, Georgia Southern University, Statesboro, GA 30460, USA; bl03096@georgiasouthern.edu (B.L.); sopoku@georgiasouthern.edu (S.T.O.); 2 Department of Biostatistics, Epidemiology, and Environmental Health Sciences, Jiann-Ping Hsu College of Public Health, Georgia Southern University, Statesboro, GA 30460, USA; jyin@georgiasouthern.edu

**Keywords:** health belief model, hotels, water safety plans, *Legionella*, microbial contamination

## Abstract

Water management plans (WMPs), sometimes referred to as risk management plans (RMPs) or water safety plans (WSPs), are not mandatory for hotels in many countries of the world, including the US. As such, many hotel personnel are uninformed of WMPs and the precautions to take if their hotel water system is compromised. The purpose of this study was to identify hotel personnel’s knowledge and practices of WMPs through a survey incorporating the Health Belief Model (HBM). Data were collected from 59 hotels within Fulton County, Georgia, USA, through a questionnaire, and questions were developed tailored to the HBM. Significant associations were found between the perceived susceptibility of contracting a waterborne illness and WMP for hotel personnel as well as between cues to action and having a WMP in general linear models (*p* ≤ 0.05). The study concludes that many key personnel are not aware of WMPs. Many hotel facilities do not have a plan in place, and some facilities are unaware of a current plan is in place. The study findings provide insight into the importance of WMPs and the risk factors associated with microbial contamination in a hotel building’s plumbing system. Future research and potential law change should be emphasized to increase hotel employees’ and owner’s WMP knowledge.

## 1. Introduction

Water safety refers to the availability of good and affordable drinking water. Water management plans (WMPs) or water safety plans (WSP), which were first introduced by the World Health Organization (WHO), are aimed at ensuring safety and decrease in contamination of water supply in both developing and developed countries [1,2]. These plans have become necessary, especially in developed countries, due to recent incidences of water contamination [3]. Currently, there are no mandatory guidelines or procedures for hotels and tourist facilities in the US and many countries of the world if the facility’s water system has been compromised by microbial contamination.

Extensive piping lines and complex plumbing in water systems of hotels may result in variability of temperature and stimulate biofilm accumulation, which collectively may favor the growth and proliferation of opportunistic and pathogenic microorganisms, including *Legionella* spp. [4,5]. These plumbing-related microorganisms can pose a serious public health risk. Several previous studies have reported *Legionella* contamination in hotel water distribution systems. For example, a study conducted in Greece reported that 20.8% of the water distribution systems in 385 Greek hotels were positive for *Legionella* spp. [6]. Another study from Southwestern Greece reported the presence of *Legionella* in 36% of water distribution systems in hotels [7]. Two other studies conducted in Italy observed percentages of *Legionella* contamination ranging from 63.6% [8] to 75% [5] in 11 and 40 Italian hotels, respectively. These studies considered culture-based methods for *Legionella* detection. When PCR-based methods were used, 74% of 19 Italian hotels showed positive results for *Legionella* contamination in their water, and the authors of this study demonstrated that culture-based methods could underestimate the contaminations [9]. A recent study of 2020 investigated the prevalence of *Legionella* contamination and its molecular diversity in 168 hotels and resorts across Israel [10] and found that out of 2830 samples tested, 470 (17%) obtained from 102 different premises (60% of hotels) were positive for *Legionella* spp. The most frequently contaminated water sources in this study were cooling towers (38%), followed by faucets, hot tubs, water lines, and storage tanks (14–17% each).

Travel-related *Legionella* cases and transmissions in hotels were reported previously [11]. Travel-associated Legionnaires’ disease was reported in more than 20 European countries, including the United Kingdom, Italy, France, and the Netherlands [12]. Travel-associated *Legionella* cases were also reported in the United States [11,13,14,15], Canada, and Australia [16,17]. Barskey et al. [15] recently reported that out of 12,200 Legionnaires’ disease cases reported among US residents between 2015 and 2016, 12.3% were travel-associated. Rasheduzzaman et al. [18] reviewed the hotel and non-hotel-associated Legionnaires’ disease in Europe from 2000 to 2010 and found that out of a total of 7974 reported travel-associated Legionnaires’ disease by different researchers, 7869 cases were hotel-associated, which represent about 99% of the reported travel-associated cases. All these findings underscore the importance of a WMP for controlling *Legionella* infections in hotels and tourist facilities.

In the US state of Georgia, the Georgia Department of Public Health (DPH) has recommendations for tourist accommodations after there was a *Legionella* report [19]. They have an immediate *Legionella* reporting contact (1-866-PUB-HLTH or 1-866-782-4584) for healthcare facilities, but no such hotline is available for hotels and tourist facilities. The DPH developed the Georgia Legionellosis control and investigation manual, which is used for investing an outbreak. Moreover, the DPH states that the health authority should provide the facility with educational material about the prevention of *Legionella* for tourist accommodations, including the Centers for Disease Control and Prevention (CDC) Water Management Program Toolkit [19]. The facility should implement a water management plan to prevent *Legionella*, per CDC recommendations. If the facility had a WMP in place, they should review the plan and revise it as needed [19]. However, many hotel owners, managers, and employees are not aware of what precautions to take if their hotel water system is compromised. This lack of knowledge can hinder their proactive roles in helping to raise awareness about the water quality standards and implementing these standards in Georgia hotels and tourist accommodations.

This study was intended to identify the gaps between limited knowledge of WMPs among hotel employees and the increased need for water safety plans for hotel facilities, which can ensure a safe environment for the public. The purpose of this study was to assess hotel employee knowledge on risk management procedures and risk factors for potential microbial contamination of Georgia hotel water distribution systems through a questionnaire survey incorporating the Health Belief Model (HBM) [20], which is one of the most widely used psychosocial approaches to explaining health-related behavior.

## 2. Materials and Methods

### 2.1. Sample and Population

The target population in this study includes all hotel facilities permitted through the Georgia Department of Public Health, in the Fulton County Health District. Fulton County is the most populous county of the state of Georgia, and its county seat is Atlanta, the state capital. A simple random sampling method was conducted to obtain a sample of 150 out of 260 hotels within Fulton County. To qualify for this study, the tourist accommodation facility must be permitted through the Georgia Department of Public Health. Participants must be employed by the respective facility. Data collection was accomplished through the means of survey questionnaires. These surveys were conducted face-to-face to help to reduce non-response. The reiteration of confidentiality was stressed to the staff to help to reduce the risk of response bias. The researcher was present to administer the questionnaire and to clarify any questions the participants had regarding the Survey.

### 2.2. Instrumentation

A 5-point Likert scale was used in the Survey, as the format is “often used in hospitality and tourism studies and tends to be favored over more complex scales in mail surveys” [21]. The authors of this research developed the survey. The study survey was formulated using validated questionnaires that used the HBM as an instrument to assess workers beliefs about using personal protective equipment [22] questionnaires that used for the implementation of the environmental management system [23], and a survey that addressed the attitudes of hotel managers in regard to environmental management [24].

The measurement scale consisted of the following: 1 = Strongly Disagree, 2 = Disagree, 3 = Neutral, 4 = Agree, 5 = Strongly Agree. Background information for the corresponding hotels, such as the size, ownership, facility type, and other relevant information were requested. Questionnaires were sent to 1 of 4 personnel: (1) the owner; (2) the maintenance engineer, (3) the lead housekeeper, and (4) the lead manager of each hotel. These are the personnel who are usually involved in planning and implementing WMPs in a hotel.

The HBM was used in this study to assess hotel employees’ attitudes and knowledge toward water management plans to get a better understanding of what barriers are in place. The Survey consisted of 2 sections. The 1st section consisted of general questions that pertain to the specific characteristics of the tourist accommodation, such as the age of the structure, type of facility (hotel, RV park, Bed and Breakfast, etc.), and general maintenance knowledge and attitudes. The next section consisted of questions tailored to the HBM [21]. Questions were developed to assess the six constructs of the HBM. These constructs include perceived susceptibility, perceived severity, perceived benefits, perceived barriers, cues to action, and self-efficacy. The Survey comprised 49 total questions.

### 2.3. Data Collection

Data were gathered over a three-month period (November 2019–January 2020). The researchers recruited hotel facilities via phone, email, and facility visits. Potential participants were provided a consent form that explained the purpose of the Survey along with reinsurance of confidentiality and the Survey being completely voluntary. If hotel personnel agreed, surveys were administered at the hotel location that was selected to participate. All data were collected specifically for research purposes (through our university Institutional Review Board approved protocol) and identifiable by study ID number only.

### 2.4. Data Analysis

Statistical software that was used for data analysis was the Statistical Analysis System–University Edition program, 2020 (SAS). To measure internal consistency and reliability, the Cronbach’s alpha data was used. The Chi-square test was used to analyze categorized variables. The statistical significance was shown with *p*-values less than or equal to 0.05.

## 3. Results

### 3.1. Descriptive Analysis of Data

In total, 50 out of 150 hotel facilities participated in this study, equating to a 33% response rate. All the participants were classified as hotel–motel establishments. In total, 3 out of the 50 facilities stated that their facility had experienced a waterborne outbreak. The age of the establishments in the sample size consisted as follows: 4% were 0–10 years of age, 24% were aged 11–15, 12% were 16–20, 36% were 21–30, and 24% were aged 31 and older.

Room capacity was assessed for each hotel establishment. This variable helps to gauge the size of each hotel. No hotels that participated had a room capacity of 0–25 rooms. In total, 6% of hotels had a room capacity of 26–50 rooms, 40% had a capacity of 51–75 rooms, 34% with a capacity of 76–100, and 20% of the facilities had rooms more than 100. Participants within the study consisted of four occupancies: owner (6%), manager (42%), lead engineer (34%), and lead housekeeper at 18%.

### 3.2. Knowledge of Water Management Plans (WMP) among Participants

According to collected data, 44% stated that they are not aware of a WMP, and 56% stated “yes”. Next, 34% responded with “yes” to if their respective facility has a WMP, while 44% answered “no” and 22% “did not know”. During the event of a water outage, half of the participants stated that their facility has emergency guidelines in place for such events, and 16% stated that they “did not know” and 18% stated “no”. An encouraging 92% of the respondents replied “yes” when asked if they had heard of a Boiled Water Advisory. All these responses are presented in Table 1.

It is imperative to have a designated staff member to ensure risk factors are limited or removed when monitoring a water system for a building. For the participants, 28% responded “no”, 66% answered “yes”, and 6% responded “don’t know” to if their facility has designated personnel/staff for routine maintenance of your buildings’ water system.

Cooling towers, decorative fountains, swimming pools, and spas have been directly linked to waterborne outbreaks when these items have been neglected and or poorly maintained. Only 6% of the hotels had cooling towers. Only 28% of the hotels had decorative fountains. Most of the hotels had a swimming pool at their facility (78%). In conjunction, 58% of the hotels had a spa that is not drained between use. During non-vacation seasons, many hotels have rooms that are not occupied for an extended period; 30% of the hotels stated that they had rooms that are unoccupied for an extended period during their “slow seasons”, which could be a risk factor for bacterial growth in the premise plumbing fixtures [25].

### 3.3. Health Belief Model Data Analyses

Section 2 of the Survey consisted of questions that are tailored to the constructs of the HBM. Participants were given five possible selections to choose on their beliefs toward specific topics. The options were strongly agree, agree, neither, disagree, and strongly disagree.

#### 3.3.1. Perceived Susceptibility

The first construct was perceived susceptibility. The participants were asked five questions to assess their susceptibility to a waterborne illness. Ten percent believed the chances of a waterborne outbreak occurring at their facility are high. Twenty-four stated that they agreed and or strongly agreed that they worry about their guests and staff contracting a waterborne illness at their facility. Participants were asked if they felt that there is a good chance of getting a waterborne illness during their career. Twelve percent either agreed or strongly agreed. Lastly, 60% of the participants agreed or strongly agreed that a waterborne illness could be prevented by developing proper maintenance procedures for their facilities’ water/plumbing system. See Table 2 for further detail.

#### 3.3.2. Perceived Severity

Hotel worker participants answered questions pertaining to their beliefs of severity in relation to a waterborne illness outbreak that their facility. In response to the first question asked, “The thought of my hotel causing a waterborne outbreak concerns me,” 74% agreed or strongly agreed to that statement. Forty-eight percent strongly agreed that if a waterborne illness occurred at their facility, the facility’s reputation would be ruined. Fifty-two percent believed that their financial security would be ruined if an outbreak occurred at their facility. See Table 3 for further detail.

#### 3.3.3. Perceived Benefits

To gauge how participants felt they would benefit from having a WMP, a series of questions was asked. In response to the question, “Having a water management plan will help reduce the risk of a waterborne outbreak from occurring,” 70% agreed and strongly agreed. Next, 84% agreed and strongly agreed that having a water management plan ensures that their staff and guests are not exposed to waterborne contaminants. Lastly, 70% of the participants agreed that a WMP would be beneficial to their facility. See Table 4 for results.

#### 3.3.4. Perceived Barriers

Barriers to the implementation of WMP were assessed. When involving staff, 58% claimed to not have enough staff to implement and maintain a water safety plan. Furthermore, 64% agreed that having a WMP is time-consuming. For additional current resources, 68% agreed that they lack knowledge of the American Society of Heating, Refrigerating and Air-Conditioning Engineers (ASHRAE) Standard 188 and the CDC Water Management Plan Toolkit. In relation to enforcement, 66% of the participants stated that they do not have a WMP because it is not mandatory. When asked if the lack of financial support for maintaining a WMP was an issue, 52% agreed. In total, 62% agreed that lack of training for staff to maintain a WPM was an issue. Seventy-two percent agreed that the lack of explanation of concepts and the need for more guidance on the public health aspect of water safety plan were a barrier. See Table 5 for results.

#### 3.3.5. Cues to Action

To assess what triggers are needed for the decision-making process to start the implementation of a WMP, questions for cues to action were asked to the participants. See Table 6 below. The following percentages are what participants stated they agreed/strongly agreed with. Receiving more encouragement from the local health authority to implement a water safety plan is important (86%). Regular and frequent education on the importance of water safety plans will help with implementation (72%). Having a simple method to implement a water safety plan will increase my chances of maintaining one (90%). Provided training will encourage our facility to implement water safety plans (86%). I am interested in water safety plans because I do not want my staff and guest exposed to any waterborne diseases (90%).

#### 3.3.6. Self- Efficacy

Self-efficacy questions were asked to the participants to gauge the level of maintaining a WMP at their respective facilities. Participants agreed to the following questions: Once I receive more education on water safety plans, I will be more comfortable in implementing the program (80%). The results can be seen below in Table 7.

### 3.4. Relationship between Hotel Facilities with no WMPs and Perceived Susceptibility to Waterborne Illness

The general linear regression model was used to assess if there was a significant relationship between whether hotel facilities with or without WMP and perceived susceptibility of contracting a waterborne illness in a hotel establishment. A significant relationship was found between the perceived susceptibility of contracting a waterborne illness and WMP for hotel personnel, with the *p*-value resulting in less than 0.05. In this event, we can positively conclude that having no WMP is associated with the workers’ perceived susceptibility to a waterborne illness (Table 8). Compared to the baseline response, respondents who have a WMP are associated with larger perceived susceptibility scores, while respondents who answered “don’t know” are not significantly different from the reference group with a *p*-value of 0.43 greater than 0.05.

### 3.5. Correlation between Cues to Action and Implementation of WMPs

The general linear model was used to analyze the relationship between cues to action and the implementation of having a WMP. A significant association was determined between cues to action and having a WMP, with a *p*-value less than 0.05. Increased cues to action are associated with an increased likelihood of WMP implementation (Table 9).

The “NO” response is set as the comparison group; the “don’t know” response is not statistically significant because its *p*-value (0.7559) is greater than the significance level of 0.05. However, there is a statistically significant association between cues to action and hotel respondents with a WMP, having a *p*-value of 0.0025.

### 3.6. Perceived Benefits and Barriers Identified by Hotel Workers for Implementing WMPs

Over 70% of the respondents responded favorably to each perceived benefit question in the questionnaire. When combining “disagree” with “strongly disagree” results and “agree” with “strongly agree” results in the barrier questions, over 50% agreed with seven out of the eight barriers.

Finally, the internal consistency and reliability of survey questions were checked using the Cronbach Alpha Reliability test. The summative scores for each scale are provided in Table 10.

## 4. Discussion

There are several agencies that have developed resources and guidelines for WMPs in the US and abroad. Such agencies as the CDC, ASHRAE, and the World Health Organization (WHO) developed basic standards to help building owners to be protected from the risk of microbial contamination. Owners and managers of hotels and tourist accommodations with high biofilm risk complex plumbing systems should use WMPs to prevent the growth and spread of *Legionella*, and they should have adequate knowledge about these WMPs. However, according to this study, 68% of the respondents lack an understanding of what these agencies provide. With the lack of awareness of available resources and the general understanding of WMPs, this could also be a major contributor to having no WMP. These findings are closely similar to a study conducted by Chan [21], where the study aimed to investigate barriers to implement environmental management systems (EMS) in the hotel industry in Hong Kong. Similar to this study, lack of knowledge, lack of professional advice, lack of resources, and cost were identified in this study as barriers. The identified barriers also align with another similar study conducted in the UK [23]. These similar findings help to strengthen the need for further research to combat these barriers from the implementation of WMPs.

Our findings indicate that regardless of the size of the building, water systems should have some type of water management plan to reduce the risk of water contamination through their plumbing system. Each facility must develop a WMP that is tailored to that specific building. Key hotel personnel who are responsible for implantation must consider (1) understanding the building’s plumbing system, (2) identifying all areas of the plumbing endpoints, (3) ensuring all water equipment and fixtures are maintained and kept in a good state, (4) developing teams that are responsible for reducing stagnation of water, biofilm and mold buildup at water fixtures, (5) creating a maintenance log for all cleanings, checks, and services, and (6) developing an emergency plan in case of a water emergency, such as a boil water advisory or do not drink advisory. In addition, hotel personnel should pay attention to water temperature levels because *Legionella* spp. grows well in warm water, and many previous outbreaks were associated with hot water systems [26,27].

There are other types of facilities that are required to have a WMP to prevent the risk of illness to the public. The Centers for Medicare and Medicaid Services (CMS) released a survey and certification memo in June 2017 indicating all healthcare facilities should develop and adhere to ASHRAE-compliant water management programs to reduce the risk for *Legionella* and other pathogens in their water systems [19]. We think similar regulatory approaches should be considered for hotels and tourist facilities.

Overall, this study is unique compared to other studies on WMPs, as we used the HBM to help to identify barriers and predict the change of a facility’s behavior for the implementation of a program. This approach is important due to the array of factors that are assessed not only at the broad company level but at the individual level. If behavior change is done at the induvial level, we hope to create change through an ecological effect.

There were a few possible limitations to the study. Response bias could have been a possibility for the study. Though the Survey was introduced and explained as confidential, voluntary, and for research purposes, many facilities were not comfortable with disclosing information to the researcher due to fear of job loss and negative publicity. Another limitation of the study was a low response rate of 33%.

There are limited studies pertaining to WMPs for hotel facilities. We hope that our findings can aid future studies in this area. Overall, the results of this study have determined that the vast majority of hotel personnel are not aware of water management plans. As the surveys stated, many hotel facilities would implement a WMP if they knew more about it. This study shows that if awareness for the program is provided, it is more likely for the facility to implement the program. As we enter a world forever transformed by the current pandemic, health and safety programs will no longer be an afterthought. For example, new guidelines to help to slow the spread of COVID-19 will be in place for the foreseeable future as awareness of the severity of the disease has risen. Hence, the same can be done simultaneously for waterborne illnesses in hotels and tourist facilities. We believe this study, along with many others mentioned above, will serve as a guideline to show how our hotels and other service industries can win the trust of Georgians and other American people that their health is safe.

## 5. Conclusions

A significant relationship was found between cues to action and the implementation of having a WMP and also between the perceived susceptibility of contracting a waterborne illness and WMP for hotel personnel. The study concludes that if hotel personnel believe that they are susceptible to an outbreak, they will be more likely to implement a WMP. This solidifies the need for research to continue to ensure the health of our larger communities. It was encouraging to see that many of the participants who were not aware of WMPs became interested in learning more about the program during the time of recruitment of this study. If there is no push for further research and raising awareness, people will continue to be susceptible to another waterborne outbreak while they may be vacationing with loved ones, at a conference, or other circumstances that involve hotel stays.

## Figures and Tables

**Table 1:** Awareness and guidelines against waterborne illness among hotel employees.

Item	Frequency (*N*)	Percentage (%)
**Have you heard of a WMP?**		
Yes	28	56.0
No	22	44.0
**Does your facility have a WMP?**		
Yes	17	34.0
No	22	44.0
Don’t Know	11	22.0
**Does your facility have emergency guidelines and or procedures in the event of a water outage?**		
Yes	16	32.0
No	25	50.0
Don’t Know	9	18.0
**Have you heard of a Boil Water Advisory?**		
Yes	46	92.0
No	4	8.0
**Does your facility have emergency guidelines and or procedures during an event of a Boil Water Advisory?**		
Yes	25	50.0
No	9	18.0
Don’t know	16	32.0

**Table 2:** Perceived susceptibility of waterborne illnesses among hotel employees.

Item	Response	Frequency (*N*)	Percentage (%)
I believe the chances of a waterborne outbreak occurring at my facility is great	Strongly Agree	3	6.0
	Agree	2	4.0
	Neither	18	36.0
	Disagree	14	28.0
	Strongly Disagree	14	28.0
I worry about my guest and staff getting a waterborne illness	Strongly Agree	4	8.0
	Agree	8	16.0
	Neither	10	20.0
	Disagree	13	26.0
	Strongly Disagree	15	50.0
I feel that there is a good chance of getting a waterborne illness during my career.	Strongly Agree	5	10.0
	Agree	1	2.0
	Neither	8	16.0
	Disagree	13	26.0
	Strongly Disagree	25	46.0
I know other hotel facilities that had a waterborne outbreak at their facility	Strongly Agree	9	18.0
	Agree	10	20.0
	Neither	10	20.0
	Disagree	9	18.0
	Strongly Disagree	12	24.0
I can prevent a waterborne illness by developing proper maintenance procedures for my facilities water/plumbing system	Strongly Agree	18	36.0
	Agree	12	24.0
	Neither	8	16.0
	Disagree	7	14.0
	Strongly Disagree	5	10.0

**Table 3:** Perceived severity of waterborne illnesses among hotel employees.

Item	Response	Frequency (*N*)	Percentage (%)
The thought of my hotel causing a waterborne outbreak concerns me	Strongly Agree	18	36.0
	Agree	19	38.0
	Neither	7	14.
	Disagree	4	8.0
	Strongly Disagree	2	4.0
If a waterborne illness occurs at my facility, my facility reputation would be ruined	Strongly Agree	4	2.0
	Agree	8	6.0
	Neither	10	16.0
	Disagree	13	28
	Strongly Disagree	15	48.0
Financial security will be endangered if a waterborne outbreak occurs at my facility	Strongly Agree	14	28.0
	Agree	12	24.0
	Neither	12	24.0
	Disagree	11	22.0
	Strongly Disagree	1	2.0
I believe my staff and guests could die prematurely if they contract a waterborne illness at my facility	Strongly Agree	15	30.0
	Agree	6	12.0
	Neither	6	12.0
	Disagree	12	24.0
	Strongly Disagree	11	22.0

**Table 4:** Perceived benefits of having a water management plan (WMP).

Item	Response	Frequency (*N*)	Percentage (%)
Having a water management plan will help reduce the risk of a waterborne outbreak from occurring	Strongly Agree	20	40.0
	Agree	15	30.0
	Neither	8	16.0
	Disagree	6	12.0
	Strongly Disagree	1	2.0
Having a water management plan ensures that my staff and guests are not exposed to waterborne contaminates	Strongly Agree	23	46.0
	Agree	19	38.0
	Neither	7	14.0
	Disagree	1	2.0
	Strongly Disagree	0	0.0
The implementation of a water safety plan will be beneficial to my facility	Strongly Agree	21	42.0
	Agree	14	28.0
	Neither	8	16.0
	Disagree	6	12.0
	Strongly Disagree	1	2.0

**Table 5:** Perceived barriers preventing implementation of a WMP.

Item	Response	Frequency (*N*)	Percentage (%)
I don’t have enough staff to implement and maintain a water safety plan	Strongly Agree	14	28.0
	Agree	15	30.0
	Neither	13	26.0
	Disagree	5	10.0
	Strongly Disagree	3	6.0
Implementing a water safety plan is time-consuming	Strongly Agree	18	36.0
	Agree	14	28.0
	Neither	7	14.0
	Disagree	4	8.0
	Strongly Disagree	7	14.0
Do we lack an understanding of ASHRAE standard 188 and the CDC water management plan toolkit?	Strongly Agree	19	38.0
	Agree	15	30.0
	Neither	8	16.0
	Disagree	5	10.0
	Strongly Disagree	3	6.0
I don’t have a water safety plan because it is not mandatory	Strongly Agree	14	28.0
	Agree	19	38.0
	Neither	1	2.0
	Disagree	8	16.0
	Strongly Disagree	8	16.0
We lack the financial support to maintain a water management plann	Strongly Agree	14	28.0
	Agree	12	24.0
	Neither	14	28.0
	Disagree	5	10.0
	Strongly Disagree	5	10.0
We lack training for staff to effectively maintain a water safety plan	Strongly Agree	19	38.0
	Agree	12	24.0
	Neither	8	16.0
	Disagree	6	12.0
	Strongly Disagree	5	10.0
We lack an explanation of concepts and more guidance needed on the public health aspect of the water safety plan.	Strongly Agree	21	42.0
	Agree	15	30.0
	Neither	7	14.0
	Disagree	3	6.0
	Strongly Disagree	4	8.0
There are no benefits to implement a water safety plan	Strongly Agree	6	12.0
	Agree	5	10.0
	Neither	9	18.0
	Disagree	14	28.0
	Strongly Disagree	16	32.0

**Table 6:** Cues to action for hotel employees to implement a WMP.

Item	Response	Frequency (*N*)	Percentage (%)
Receiving more encouragement from the local health authority to implement a water safety plan is important	Strongly Agree	28	56.0
	Agree	15	30.0
	Neither	5	10.0
	Disagree	2	4
	Strongly Disagree	0	0
Regular and frequent education on the importance of water safety plans will help with implemeantation.	Strongly Agree	27	54.0
	Agree	9	18.0
	Neither	12	24.0
	Disagree	1	2.0
	Strongly Disagree	1	2.0
Having a simple method to implement a water safety plan will increase my chances of maintaining one.	Strongly Agree	31	62.0
	Agree	14	28.0
	Neither	5	10.0
	Disagree	0	0.0
	Strongly Disagree	0	0.0
Provided training will encourage our facility to implement water safety plans	Strongly Agree	22	44.0
	Agree	21	42.0
	Neither	7	14.0
	Disagree	0	0.0
	Strongly Disagree	0	0.0
I am interested in water safety plans because I do not want my staff and guest exposed to any waterborne diseases	Strongly Agree	27	54.0
	Agree	18	36.0
	Neither	5	10.0
	Disagree	0	0.0
	Strongly Disagree	0	0.0

**Table 7:** Self-efficacy of hotel employees to maintain a WMP.

Item	Response	Frequency (*N*)	Percentage (%)
Once I receive more education on water safety plans, I will be more comfortable in implementing the program	Strongly Agree	20	40.0
	Agree	20	40.0
	Neither	9	18.0
	Disagree	0	0.0
	Strongly Disagree	1	2.0
I am confident that maintaining a water safety plan will help prevent a waterborne illness outbreak at my facility	Strongly Agree	20	40.0
	Agree	15	30.0
	Neither	12	24.0
	Disagree	2	4.0
	Strongly Disagree	1	2.0
I can train my staff to maintain records for water safety plans	Strongly Agree	17	34.0
	Agree	13	26.0
	Neither	13	26.0
	Disagree	6	12.0
	Strongly Disagree	1	2.0
I am confident that I can manage the additional duty of implementing a water safety plan.	Strongly Agree	16	32.0
	Agree	14	28.0
	Neither	14	28.0
	Disagree	4	8.0
	Strongly Disagree	2	4.0
It is our responsibility as hotel staff to provide safe potable water to our guests.	Strongly Agree	30	60.0
	Agree	16	32.0
	Neither	4	8.0
	Disagree	0	0.0
	Strongly Disagree	0	0.0

**Table 8:** The general linear model showing the relationship between WMP and perceived susceptibility.

Source	DF	Sum of Squares		Mean Square	*F* Value	Pr > F
Model	2	122.817		61.40	3.77	*0.0302*
Error	47	764.962		16.27		
Corrected Total	49	887.780				
**Parameter**	**Estimate**			**Standard Error**	***t* Value**	**Pr > |t|**
Don’t Know	1.18181818		B	1.48977420	0.79	0.4316
Yes	3.56149733		B	1.30276975	2.73	0.0088
NO	0.00000000		B	-	-	-

**Table 9:** The general linear model showing the relationship between WMP and cues to action.

Parameter	Estimate		Standard Error	*t* Value	Pr > |t|
Don’t Know	0.31818182	B	1.01774586	0.31	0.7779
Yes	2.84759358	B	0.88999294	3.20	0.0025
NO	0.00000000	B	-	-	-

**Table 10:** The internal consistency and reliability of survey questions checked by the Cronbach Alpha Reliability test (summative scores for scales).

Scale	Number of Items in Scale	Cronbach’s Alpha
perceived susceptibility	5	0.67
perceived severity	4	0.70
perceived benefits	3	0.88
perceived barriers	8	0.71
cues to action	5	0.77
self-efficacy	5	0.83

## Data Availability

All collected data are contained within the article.
